# Cold storage of *Gonipterus platensis* (Coleoptera: Curculionidae) eggs for *Anaphes nitens* (Hymenoptera: Mymaridae) rearing

**DOI:** 10.7717/peerj.20903

**Published:** 2026-03-13

**Authors:** Murilo Fonseca Ribeiro, Gabriela Cavallini, Gabriel Negri Solce, Ana Laura Favoreto, José Raimundo De Souza Passos, Leonardo Rodrigues Barbosa, Brett Hurley, Carlos Frederico Wilcken

**Affiliations:** 1 Instituto de Pesquisas e Estudos Florestais (IPEF), Piracicaba, São Paulo, Brazil; 2 Departamento de Proteção Vegetal/Faculdade de Ciências Agronômicas, Universidade Estadual Paulista, Botucatu, São Paulo, Brazil; 3 Departamento de Biodiversidade e Bioestatística/Instituto de Biociências, Universidade Estadual Paulista, Botucatu, São Paulo, Brazil; 4 Embrapa Florestas, Empresa Brasileira de Pesquisa Agropecuária, Colombo, Paraná, Brazil; 5 Department of Zoology and Entomology/Forestry and Agricultural Biotechnology Institute (FABI), University of Pretoria, Pretoria, Gauteng, South Africa

**Keywords:** Biological control, Egg parasitoid, Eucalyptus, Eucalyptus snout-beetle, Mass rearing

## Abstract

**Background:**

* Anaphes nitens* (Girault, 1928) (Hymenoptera: Mymaridae) is an egg parasitoid used for the biological control of *Gonipterus* spp. in regions where this pest is present. Cold storage of host eggs is a crucial strategy in biological control programs. This study aimed to evaluate the effects of cold storage on *G. platensis* eggs for laboratory rearing of both the host and its parasitoid, *A. nitens*.

**Methods:**

*Gonipterus platensis* eggs, aged 1 and 3 days, were stored in a refrigerator at 7 ± 1 °C for 5, 10, 20, 30, 40, and 50 days in complete darkness. After each storage period, the eggs were transferred to a biochemical oxygen demand (BOD) chamber at 25 °C and hatching rates and larval survival were assessed. To evaluate parasitoid reproduction, fresh host eggs were stored at 7 ± 1 °C for 5, 10, 15, 20, and 25 days under dark conditions and subsequently exposed to *A. nitens*. Parasitism rate, sex ratio, and offspring longevity were recorded.

**Results:**

Results showed that 1- and 3-day-old *G. platensis* eggs maintained the highest viability within the first 10 days of storage, with hatching rates exceeding 50% up to 20 days post-storage. A 20-day storage period was the longest duration that did not differ significantly from the non-stored eggs (control) considering *A. nitens* parasitism and progeny longevity when stored host eggs were offered.

**Conclusion:**

Therefore, storing host eggs for this period effectively supports colony maintenance while simultaneously facilitating parasitoid propagation.

## Introduction

*Gonipterus platensis* (Marelli, 1926) (Coleoptera: Curculionidae) is native to Australia and one of the most significant defoliators of *Eucalyptus* worldwide (Schröder et al., 2020). Both adults and larvae feed on young leaves and shoots, causing partial or complete defoliation of tree canopies. This damage can lead to reduced growth, branching abnormalities, or even tree mortality (Reis et al., 2012; Souza et al., 2016; Rua et al., 2020). Currently, biological control is the primary strategy used to manage these insects, with emphasis on *Anaphes nitens* (Girault,1928) (Hymenoptera: Mymaridae) (Valente et al., 2018).

*Anaphes nitens* is a solitary egg parasitoid of Australian origin, idiobiont and endoparasitic (Tooke, 1955). This parasitoid was introduced globally for the control of *Gonipterus* spp. at a time when *Gonipterus scutellatus* Gyllenhal, 1833 was believed to be the only invasive species of the genus. This genus is now recognized as a complex of at least eight species, including *G. platensis* (Mapondera et al., 2012). *Anaphes nitens* remains the primary biological control agent against *Gonipterus* spp. worldwide (Schröder et al., 2020), with new introductions still occurring to manage pest outbreaks (Barten et al., 2023). Females of *A. nitens* are weakly synovigenic and can detect fresh *G. platensis* eggs, parasitizing up to 70 host eggs over their lifetime (Santolamazza-Carbone & Cordero-Rivera, 2003a; Santolamazza-Carbone, Rodrıguez Illamola & Cordero-Rivera, 2004; Valente et al., 2017a).

The rearing of *A. nitens* must occur in *Gonipterus* spp. eggs, as these parasitoids are highly specific to their host (Valente et al., 2017b). However, rearing *Gonipterus* in the laboratory is challenging, as these insects can go several weeks without ovipositing (Ribeiro et al., 2024), potentially leading to the depletion of parasitoid colonies. To address this issue, storing *G. platensis* egg capsules at low temperatures may serve as a viable strategy for sustaining *A. nitens* production, as this strategy can extend shelf-life and reduce problems with lack of availability of hosts and parasitoids throughout the year (Barbosa et al., 2025).

The storage of host insect eggs for the rearing of Mymaridae parasitoids has been previously reported. *Homalodisca vitripennis* (Germar, 1821) (previously referred as *Homalodisca coagulate*) (Hemiptera: Cicadellidae) eggs can be stored for up to 30 days at 10 °C without detrimental effects on the parasitoid *Cosmocomoidea ashmeadi* (Girault, 1915) (previously referred as *Gonatocerus ashmeadi)* (Hymenoptera: Mymaridae) (Chen & Leopold, 2007). Similarly, the parasitoid *Cleruchoides noackae* Lin & Huber, 2007 (Hymenoptera: Mymaridae) exhibits comparable parasitism rates in both fresh and stored eggs of *Thaumastocoris peregrinus* Carpintero & Dellapé, 2006 (Hemiptera: Thaumastocoridae) when stored at 5 °C for up to 14 days (Barbosa et al., 2018).

This study aimed to determine the optimal cold storage duration at 7 °C in complete darkness for *G. platensis* eggs, supporting both their own rearing and the laboratory rearing of their egg parasitoid *A. nitens* within an augmentative biological control framework.

## Material and Methods

The methodology below describes two experiments conducted to address the objectives of this study. The first experiment evaluated whether stored eggs can be used to maintain host rearing under laboratory conditions. The second experiment assessed whether stored host eggs remain suitable for rearing the parasitoid.

### *Gonipterus platensis* rearing

The rearing of *G. platensis* was conducted in a climate-controlled room at 25 ± 1 °C, 50 ± 10% relative humidity, and a 12:12 h light-dark photoperiod.

Thirty pairs of *G. platensis* were housed in wooden cages measuring 80  × 40  × 45 cm (height × width × depth), with a glass top and voile fabric sides. Each cage contained a bouquet of *Eucalyptus urophylla* branches with tender leaves and shoots. The petiole of the bouquet was placed in 500 mL plastic containers filled with distilled water, which was replaced every two days.

The egg capsules collected from the rearing were placed in five cm diameter acrylic Petri dishes within the same room. Larval hatching was monitored daily in the morning. Once hatched, the larvae were transferred to a designated bouquet for feeding, where they remained until they reached the pre-pupal stage. At this stage, they stopped feeding and moved toward the bottom of the cage.

For pupation, 10 pre-pupae were placed in 1 L plastic containers filled with 150 g of autoclaved sand moistened with 15 mL of distilled water. They remained there until they emerged as adults.

### *Anaphes nitens* rearing

Adults of *A. nitens* were reared in flat-bottomed glass tubes (1.5 cm in diameter, 10 cm in height) sealed with voile fabric. Each tube contained two parasitoid pairs and six *G. platensis* egg capsules that were no more than 24 h old.

The parasitoids were fed pure honey and kept for three days in a biochemical oxygen demand (BOD) chamber at 20 ± 1 °C, 60 ± 10% relative humidity, and a 12:12 h light-dark photoperiod to allow for parasitism. After this period, the egg capsules were transferred to five cm diameter acrylic Petri dishes and maintained under the same conditions until the emergence of the next generation.

### Cold storage of *Gonipterus platensis* eggs

A total of 495 *G. platensis* egg capsules, less than 24 h old and obtained from laboratory rearing, were placed in five cm diameter acrylic Petri dishes and kept in BOD chambers at 25 ± 1 °C, 60% relative humidity, and a 12:12 h light-dark photoperiod (standard environmental conditions). Of these, 375 egg capsules were used for the host survival experiment, while 120 were allocated to the parasitism experiment. Each egg capsule was considered an experimental unit, meaning the number of eggs evaluated varied across treatments due to the natural variation in egg counts per capsule.

Egg capsules were maintained under standard environmental conditions for one and three days, allowing the development of immatures of different ages within the eggs. Following these initial periods, the egg capsules were separated by age (one and three-days-old) and randomly assigned into seven groups per egg age (25 capsules per group) for storage in acrylic plates in a refrigerator at 7 ± 1 °C under constant darkness for 5, 10, 20, 30, 40, 50, and 60 days. Twenty-five egg capsules of one day and not subjected to storage served as control. After the storage period, the capsules were returned to the BOD, where they remained under standard environmental conditions along with the control eggs until larval hatching.

After the storage period, a young *E. urophylla* leaf was placed inside each acrylic plate containing one egg capsule to feed the newly hatched larvae. Once hatched, larvae were transferred to 1-liter plastic containers (11 × 14 × 9 cm, height × width × depth) containing a branch of *E. urophylla*. The containers were kept under standard environmental conditions.

The number of hatched larva and larval mortality were assessed daily. After seven days, the egg capsules were dissected under a Nikon SMZ645 stereomicroscope to count any retained insects and infertile eggs, and the final mortality was calculated.

Once the maximum egg storage period for *G. platensis* was determined, with the least impact on hatching and larval survival, an experiment was set up to multiply the parasitoid *A. nitens*.

### Cold storage of *Gonipterus platensis* eggs for *Anaphes nitens* rearing

*Gonipterus platensis* egg capsules up to one-day-old were stored in refrigerators at 7 ± 1 °C under constant darkness, for different durations: 0 (non-stored control) 5, 10, 15, 20, and 25 days. Each egg capsule represented an experimental unit, and each treatment included 20 replicates.

After the storage period, each egg capsule was kept with one *A. nitens* mated female (up to 48 h old) inside a flat-bottom glass tube (1.5 cm in diameter, 10 cm in height), sealed with Parafilm^®^. The setup was maintained for 24 h in a BOD chamber at 20 ± 1 °C, 60% relative humidity, and a 12:12-hour light-dark cycle. The parasitoids were fed with pure honey.

After the parasitism period, the egg capsules were transferred to five cm diameter acrylic Petri dishes and kept under the same environmental conditions. They were monitored daily until larval hatching and/or parasitoid emergence. Ten days after the last parasitoid emerged, the egg capsules were dissected under a Nikon SMZ645 stereomicroscope to count any retained insects.

Parasitism was calculated using the following formula: parasitism rate (P) = ((number of emerged parasitoids + number of retained parasitoids)/total eggs) ×100%.

The parasitoid emergence rate was calculated using the following formula: emergence rate (E) = (number of emerged parasitoid/total of parasitoids) × 100%.

After progeny emergence, the parasitoids were individually placed in flat-bottom glass tubes (1.5 cm in diameter, 10 cm in height), fed with pure honey, and kept in a BOD chamber at 20 ± 1 °C, 60% relative humidity, and a 12:12-hour light-dark cycle to assess longevity.

### Statistical analyses

Statistical analyses were performed using generalized linear models (Nelder & Wedderburn, 1972), with variations based on distribution and link function (Table 1).

**Table 1:** Factors, distribution and link function used in the statistical analyses of each of the response variables measured for the *Gonipterus platensis* egg storage experiments.

Experiment	Response variable	Factors (covariates)	Distribution	Link function
Cold storage *G. platensis* eggs	Hatching percentage	Treatments; Egg age	Binomial	Logit
	Survival percentage	Treatments	Binomial	Logit
Cold storage for *A. nitens* rearing	Parasitism	Treatments	Gama	Logarithmic
	Emergence rate	Treatments	Binomial	Logit
	Sex ratio	Treatments	Binomial	Logit
	Progeny longevity	Treatments; Sex	Gama	Logarithmic

For all fitted generalized linear models, model quality was assessed through deviance analysis and standardized Pearson residual plots. Treatment comparisons were conducted using the Tukey-Kramer test (Westfall et al., 1999) within the *genmod* procedure of the SAS statistical software—Free Statistical Software, SAS University Edition. All data are presented as mean ± standard error (SE).

## Results

### Cold storage of *Gonipterus platensis* eggs

**Figure 1:**
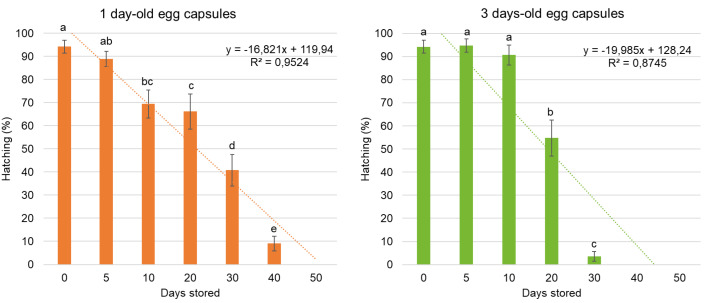
Hatching percentage (%) (mean ± SE) of *Gonipterus platensis* larvae of different egg storage periods at 7 ± 1 °C. (T = 25 ± 1 °C, relative humidity of 50 ± 10% and photoperiod 12:12h).

There was a significant interaction between cold storage duration and egg age, reflected in a higher hatching percentage of *G. platensis* from 3-day-old eggs compared with 1-day-old eggs up to 10 days of storage (*p* < 0.0001). The hatching rate of non-stored eggs was 94.25% ± 2.80%. For one-day-old eggs, the highest larval hatching rate following cold storage was observed after 5 days (88.89% ± 3.32%) which was not significantly different from the control. Eggs stored for 10 days also showed relatively high hatching rate (69.40% ± 6.07%). In contrast, one-day-old eggs stored for 40 days had the lowest hatching rate (9.06% ± 3.12%), and no hatching was observed in eggs stored for 50 days or longer. For three-day-old eggs, hatching rates after 5 days (94.81% ± 2.88%) and 10 days (90.72% ± 4.31%) of cold storage were not significantly different from the control and were higher than those of eggs stored for 30 days (3.52% ± 2.09%). No larval hatching was observed after 40 days of cold storage for three-day-old eggs (Fig. 1).

**Figure 2:**
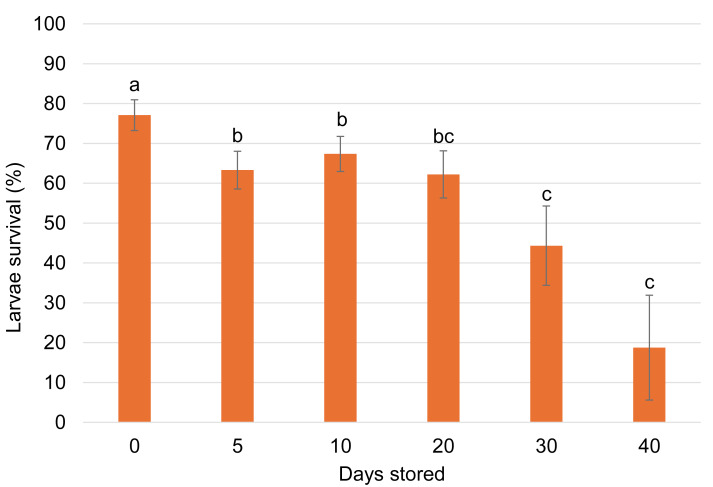
Survival rates after 7 days of hatching (%) (mean ± SE) of *Gonipterus platensis* eggs subjected to different storage periods at 7 ± 1 °C to 25 ± 1 °C.

No significant interaction was observed between egg age and cold storage duration on the survival of *G. platensis* larvae seven days after hatching (*p* = 0.2351). As a result, data were pooled and analyzed based on storage duration alone. Larval survival was significantly higher in the control group (77.08% ± 3.86%) than in any of the storage-duration treatments. Survival among treatments stored for 5 to 20 days did not differ significantly and ranged from 62.22% ± 5.92% to 67.35% ± 4.40% (Fig. 2).

### Cold storage of *Gonipterus platensis* eggs for *Anaphes nitens* rearing

**Figure 3:**
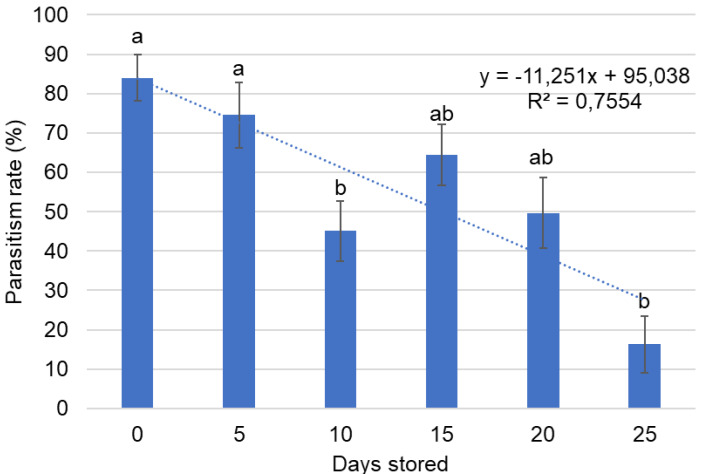
Parasitism (Mean ± SE) of *Anaphes nitens* in *Gonipterus platensis* eggs subjected to different storage periods at 7 ± 1 °C. Means followed by the same letter do not differ according to the Tukey-Kramer test (*p* < 0.05).

The cold storage periods of *G. platensis* eggs affected *A. nitens* parasitism (*p* = 0.0145). The parasitism rate was highest (74.54% ± 8.34%) and lowest (16.31% ± 7.22%) in eggs stored at cold temperatures for 5 and 25 days, respectively (Fig. 3).

Cold storage periods did not significantly influence pre-emergence survival (parasitoids successfully completing development inside the eggs) (*p* = 0.7294), with values varying from 71.67 ± 18.56% to 91.76 ± 6.20% (Table 2). Similarly, the sex ratio progeny was not affected by storage duration (*p* = 0.4796), which ranged from 0.58 ± 0.16 to 0.74 ± 0.06 (Table 2).

**Table 2:** Longevity of adults (Long. Progeny) (Mean ± SE) and sex ratio of progeny (sr) (Mean ± SE) of *Anaphes nitens* from host eggs of *Gonipterus platensis* subjected to different storage periods at 7 ± 1 °C. Longevity of adults were calculated adding males and females.

Storage time (days)	Parasitoid emergence rate (%)	Long. Progeny (days)	sr
0	87.15 ± 4.89 a	7.99 ± 0.52 b	0.60 ± 0.04 a
5	91.76 ± 6.20 a	8.04 ± 0.47 ab	0.74 ± 0.06 a
10	92.70 ± 4.01 a	8.37 ± 0.89 ab	0.65 ± 0.09 a
15	80.09 ± 6.32 a	11.60 ± 0.69 a	0.65 ± 0.06 a
20	82.74 ± 8.03 a	9.76 ± 0.84 ab	0.59 ± 0.07 a
25	71.67 ± 18.56 a	10.00 ± 1.39 ab	0.58 ± 0.16 a

**Notes.**

Means followed by the same letter do not differ according to the Tukey-Kramer test (*p* < 0.05).

There was no interaction between storage time and the longevity of *A. nitens* progeny by sex for individuals emerging from *G. platensis* eggs stored at cold temperatures for different durations (*p* = 0.4643). Consequently, male and female data were pooled and analyzed according to storage-time factor. The average longevity of *A. nitens*, was highest for insects emerging from eggs stored for 15 days (11.60 ± 0.69), and lowest for those emerging from non-stored eggs (7.99 ± 0.52) (Table 2).

## Discussion

Augmentative biological control currently represents the main strategy to control *G. platensis* in countries where the pest is present, as Spain, Portugal, and Brazil (Ayuga-Téllez et al., 2022; Rua et al., 2020; Soliman et al., 2025). This study contributes to the management of *G. platensis* by providing information on how to maintain a laboratory colony of the pest with a constant supply of eggs, a requirement for the efficient mass rearing of the parasitoid *A. nitens*. In Brazil, early-season *A. nitens* releases have achieved parasitism rates of 80–100% in the field within 30 days after release. However, parasitism declines and becomes more variable over time, ranging from 27–86% at 120 days, indicating the need for continuous releases of the parasitoid to maintain effective control (Soliman et al., 2025).

When evaluating egg storage durations for *A. nitens* propagation, it is also important to assess potential impacts on the host colony. The results for stored eggs aged 1 and 3 days showed the highest hatching rates within the first 10 days, though hatching rates above 50% were still observed up to 20 days of storage. A 20-day storage period was also the longest duration that showed no statistical difference in *A. nitens* parasitism compared to the control. Thus, storing *G. platensis* eggs at 7 °C for up to 20 days effectively maintains the host colony while also supporting the multiplication of the parasitoid.

*Gonipterus platensis* eggs could be stored for up to 10 days without significantly affecting larval hatching rates, which began to decline after 20 days of storage. No larvae survived to hatching beyond 40 days for three-day-old eggs or after 50 days in freshly laid eggs. The minimum temperature threshold for *G. platensis* development has been estimated at 5–6.5 °C in *Eucalyptus globulus* (Santolamazza-Carbone, Rodríguez-Illamola & Cordero-Rivera, 2006) and may vary depending on the host plant, with values of 10.3–10.6 °C reported for *E. urophylla* and *E. grandis* × *E. urophylla* clones (Oliveira, 2006). Storage at 7 °C did not prevent embryonic development, allowing these eggs to be used for maintaining the host colony or multiplying the parasitoid.

The egg stage is one of the most temperature-sensitive immature stages (Maharjan et al., 2017). For *Diabrotica virgifera virgifera* LeConte, 1868 (Coleoptera: Chrysomelidae), eggs could be stored for up to two weeks without reducing hatching rates, although larval development was negatively affected (Geisert, Ludwick & Hibbard, 2019). In this study, although the full developmental period of *G. platensis* larvae was not assessed, no differences in larval survival rates were observed up to the seventh day for eggs stored for up to 20 days. This suggests that storage for this duration does not negatively impact on the insect.

The parasitism results indicate that *G. platensis* eggs stored for up to 20 days can be used for the multiplication of *A. nitens* without a significant reduction in parasitism rates. The consistency of high emergence rates of parasitoids among storage durations reinforces this proposal, indicating that cold storage within this period does not impair *A. nitens* development or emergence. Storage beyond this period using the method applied on this experiment is not recommended, as reduced temperature and/or extended exposure to low temperatures can cause cumulative and irreversible damage to parasitoids (Colinet & Hance, 2010). The larval stages of *A. nitens* feed on the host egg (Tooke, 1955), and longer storage times reduce the eggs’ nutritional value due to deterioration (Pratissoli et al., 2003), water loss, or fungal growth (Chen & Leopold, 2007).

In *A. nitens*, no change in the sex ratio of progeny was observed after parasitism in eggs stored for up to 25 days. This species naturally exhibits a female-biased sex ratio (Hanks et al., 2000; Santolamazza-Carbone & Cordero Rivera, 2003b; Barten et al., 2023), which contributes to its effectiveness in biological control programs. Maintaining this characteristic after exposure to cold stored eggs is desirable, as shifts in sex ratio could negatively affect the efficacy of pest control in the field. Low temperatures commonly alter the sex ratio of progeny in species where one sex is more susceptible to temperature-related changes in the host, leading to higher mortality of parasitoids during the larval stage (Forouzan et al., 2018). However, there are reports of species where low temperatures do not affect the sex ratio (Foerster & Doetzer, 2006), and also instances where the sex ratio only changes after long periods of host egg storage, as in *Trichogrammatoidea bactrae* Nagajara, 1979 (Hymenoptera: Trichogrammatidae), where the sex ratio only changes after 8 weeks of storage at 4 °C (Mohamed & El-Heneidy, 2020), and *Anastatus fulloi* Sheng & Wang, 1977 (Hymenoptera: Eupelmidae), where host egg storage for 12 months is required to alter the sex ratio (Zhao et al., 2021). Thus, the results obtained in this experiment for *A. nitens* are dependent on the duration of storage time of *G. platensis* host eggs.

Several studies have demonstrated that parasitism rates decrease as storage duration increases (Chen & Leopold, 2007; Spínola-Filho et al., 2014; Haque et al., 2021). In the *G. platensis–A. nitens* system, both host egg hatching and parasitism rates decreased with prolonged storage, accompanied by greater data variability. These results indicate that storage has a negative impact initiating after only 10 days for 1-day-old *G. platensis* eggs. Although a maximum storage period of 20 days may appear limited, this threshold reflects the methodological balance between optimizing parasitoid production and maintaining host quality. If the focus were solely on improving parasitoid mass rearing, alternative storage techniques could be tested, such as individual quick freezing associated with cold storage (Wu et al., 2024), cryopreservation (Ramos et al., 2025), the use of sterilized host eggs (Boly et al., 2025), or storage of parasitized eggs (Haque et al., 2021).

## Conclusion

The results indicate that *G. platensis* eggs, both one and three-days-old, can be stored at 7 °C for up to 10 days while maintaining high hatching viability. Larval survival following cold storage remained unaffected up to 20 days. Additionally, *G. platensis* eggs stored at 7 °C for up to 20 days showed no significant differences in *A. nitens* parasitism compared to the control, confirming their suitability for use in parasitoid rearing programs.

##  Supplemental Information

10.7717/peerj.20903/supp-1 Supplemental Information 1 Raw data

10.7717/peerj.20903/supp-2 Supplemental Information 2 Codebook

## Additional Information and Declarations

### Data Availability

The following information was supplied regarding data availability: The raw data are available in the Supplemental File.

## References

[ref-1] Ayuga-Téllez E, García-Iruela A, Rielo JC, González-García C (2022). Actions for monitoring the Gonipterus pest in Eucalyptus on the Cantabrian coast. Agronomy.

[ref-2] Barbosa LR, Rodrigues ÂP, De Souza LN, Foerster LA, De Souza AR, De Castroe Castro BM, Wilcken CF, Zanuncio JC (2018). Development of *Cleruchoides noackae*, an egg-parasitoid of *Thaumastocoris peregrinus*, in eggs laid on different substrates, with different ages and post-cold storage. BioControl.

[ref-3] Barbosa LR, Wilcken CF, Bush S, Zanuncio JC (2025). Rearing parasitoids for biological control programmes in plantation forests. Biological control of insect pests in plantation forests.

[ref-4] Barten H, Schröder ML, Slippers B, Howe AG, Lawson SA, Hurley BP (2023). Reproductive compatibility of a newly imported Australian population of the biocontrol agent *Anaphes nitens* with an existing South African population. Biological Control.

[ref-5] Boly A, Waongo A, Kaboré A, Traore F, Ba MN, Sanon A (2025). Comparative evaluation of two methods for sterilizing *Corcyra cephalonica* Stainton (Lepidoptera: Pyralidae) eggs for mass rearing of *Trichogrammatoidea armigera* Nagaraja (Hymenoptera: Trichogrammatidae). Phytoparasitica.

[ref-6] Chen WL, Leopold RA (2007). Progeny quality of *Gonatocerus ashmeadi* (Hymenoptera: Mymaridae) reared on stored eggs of *Homalodisca coagulata* (Hemiptera: Cicadellidae). Journal of Economic Entomology.

[ref-7] Colinet H, Hance T (2010). Interspecific variation in the response to low temperature storage in different aphid parasitoids. Annals of Applied Biology.

[ref-8] Foerster LA, Doetzer AK (2006). Cold storage of the egg parasitoids *Trissolcus basalis* (Wollaston) and *Telenomus podisi* Ashmead (Hymenoptera: Scelionidae). Biological Control.

[ref-9] Forouzan F, Jalali MA, Ziaaddini M, Hashemi Rad H (2018). Effect of cold storage on biological traits of *Psix saccharicola* (Hymenoptera: Platygastridae), an egg parasitoid of *Acrosternum arabicum* (Hemiptera: Pentatomidae). Journal of Economic Entomology.

[ref-10] Geisert RW, Ludwick DC, Hibbard BE (2019). Effects of cold storage on nondiapausing eggs of the western corn rootworm (Coleoptera: Chrysomelidae). Journal of Economic Entomology.

[ref-11] Hanks LM, Millar JG, Paine TD, Campbell CD (2000). Classical biological control of the Australian weevil Gonipterus scutellatus (Coleoptera: Curculionidae) in California. Environmental Entomology.

[ref-12] Haque A, Islam S, Bari A, Hossain A, Athanassiou CG, Hasan M (2021). Cold storage-mediated rearing of *Trichogramma evanescens* Westwood on eggs of *Plodia interpunctella* (Hübner) and *Galleria mellonella* L. PLOS ONE.

[ref-13] Maharjan R, Yi H, Young Y, Jang Y, Kim Y, Bae S (2017). Effects of low temperatures on the survival and development of *Callosobruchus chinensis* (L.) (Coleoptera: Bruchidae) under different storage durations. Journal of Asia-Pacific Entomology.

[ref-14] Mapondera TS, Burgess T, Matsuki M, Oberprieler RG (2012). Identification and molecular phylogenetics of the cryptic species of the *Gonipterus scutellatus* complex (Coleoptera: Curculionidae: Gonipterini). Austral Entomology.

[ref-15] Mohamed HO, El-Heneidy AH (2020). Effect of cold storage temperature on quality of the parasitoid, Trichogrammatoidea bactrae Nagaraja (Hymenoptera: Trichogrammatidae). The Egyptian Journal of Biological Pest Control.

[ref-16] Nelder JA, Wedderburn RW (1972). Generalized linear models. Journal of the Royal Statistical Society.

[ref-17] Oliveira NCD (2006). Biologia de *Gonipterus scutellatus* (Coleoptera: Curculionidae) em *Eucalyptus* spp. em diferentes temperaturas. Tese.

[ref-18] Pratissoli D, Vianna UR, Oliveira HN, Pereira FF (2003). Effect of storage of *Anagasta kuehniella* (Lepidoptera: Pyralidae) eggs on the biological characteristics of three Trichogramma (Hymenoptera: Trichogrammatidae) species. Ceres Journal.

[ref-19] Ramos GS, Hayashida R, Ikuno PHP, Carvalho VRd, Hoback WW, Oliveira RCd (2025). Quality assessment and host preference of Telenomus podisi (Hymenoptera: Scelionidae) for fresh and cryopreserved *Euschistus heros* (Hemiptera: Pentatomidae) eggs. Insects.

[ref-20] Reis AR, Ferreira L, Tomé M, Araujo C, Branco M (2012). Efficiency of biological control of *Gonipterus platensis* (Coleoptera: Curculionidae) by *Anaphes nitens* (Hymenoptera: Mymaridae) in cold areas of the Iberian Peninsula: implications for defoliation and wood production in *Eucalyptus globulus*. Forest Ecology and Management.

[ref-21] Ribeiro MF, Cavallini G, Solce GN, Favoreto AL, Passos JRDS, Hurley B, Wilcken CF (2024). Polyandry contributes to *Gonipterus platensis* (Coleoptera: Curculionidae) rearing. PeerJ.

[ref-22] Rua JC, Barreiro S, Branco M, Tomé M (2020). Estimating defoliation impact of *Gonipterus platensis* on *Eucalyptus globulus* stands productivity using a forest simulator based on 3-PG. Forest Ecology and Management.

[ref-23] Santolamazza-Carbone S, Cordero-Rivera A (2003a). Egg load and adaptive superparasitism in *Anaphes nitens*, an egg parasitoid of the *Eucalyptus* snout-beetle *Gonipterus scutellatus*. Entomologia Experimentalis et Applicata.

[ref-24] Santolamazza-Carbone S, Cordero Rivera A (2003b). Superparasitism and sex ratio adjustment in a wasp parasitoid: results at variance with Local Mate Competition?. Oecologia.

[ref-25] Santolamazza-Carbone S, Rodrıguez Illamola A, Cordero-Rivera A (2004). Host finding and host discrimination ability in *Anaphes nitens* Girault, an egg parasitoid of the *Eucalyptus* snout-beetle *Gonipterus scutellatus* Gyllenhal. Biological Control.

[ref-26] Santolamazza-Carbone S, Rodríguez-Illamola A, Cordero-Rivera A (2006). Thermal requirements and phenology of the *Eucalyptus* snout beetle *Gonipterus scutellatus* Gyllenhal. Journal of Applied Entomology.

[ref-27] Schröder ML, Slippers B, Wingfield MJ, Hurley BP (2020). Invasion history and management of Eucalyptus snout beetles in the *Gonipterus scutellatus* species complex. Journal of Pest Science.

[ref-28] Soliman EP, Zauza EAV, Domingues MM, Hall KB (2025). Augmentative biological control in plantation forests: a case study from a forestry company in Brazil. Biological control of insect pests in plantation forests.

[ref-29] Souza NM, Junqueira LR, Wilcken CF, Soliman EP, Camargo MB, Nickele MA, Barbosa LR (2016). Ressurgência de uma antiga ameaça: Gorgulho-do-eucalipto *Gonipterus platensis* (Coleoptera: Curculionidae). Circular Técnica IPEF.

[ref-30] Spínola-Filho PRDC, Leite GLD, Soares MA, Alvarenga AC, Paulo PDD, Tuffi-Santos LD, Zanuncio JC (2014). Effects of duration of cold storage of host eggs on percent parasitism and adult emergence of each of ten *Trichogrammatidae* (Hymenoptera) species. Florida Entomologist.

[ref-31] Tooke FGC (1955). The eucalyptus snout beetle, Gonipterus scutellatus Gyll., a study of its ecology and control by biological means.

[ref-32] Valente C, Afonso C, Gonçalves CI, Alonso-Zaragaza MA, Reis A, Branco M (2017b). Environmental risk assessment of the egg parasitoid *Anaphes inexpectatus* for classical biological control of the *Eucalyptus* snout beetle, Gonipterus platensis. BioControl.

[ref-33] Valente C, Gonçalves CI, Monteiro F, Gaspar J, Silva M, Sottomayor M, Paiva MR, Branco M (2018). Economic outcome of classical biological control: a case study on the Eucalyptus snout beetle, *Gonipterus platensis*, and the parasitoid *Anaphes nitens*. Ecological Economics.

[ref-34] Valente C, Gonçalves CI, Reis A, Branco M (2017a). Pre-selection and biological potential of the egg parasitoid *Anaphes inexpectatus* for the control of the *Eucalyptus* snout beetle, *Gonipterus platensis*. Journal of Pest Science.

[ref-35] Westfall PH, Tobias RD, Rom D, Wolfinger RD, Hochberg Y (1999). Multiple comparisons and multiple tests using the SAS system.

[ref-36] Wu YH, Lee SY, Chuang YY, Tzeng HY, Lai JS, Chou MY (2024). Effect of individual quick freezing treatment and cold storage on the host egg (Lepidoptera: Saturniidae) quality for the production of the parasitoid *Anastatus japonicus* Ashmead (Hymenoptera: Eupelmidae). Egyptian Journal of Biological Pest Control.

[ref-37] Zhao C, Zhang B, Liu Z, Zhang H, Li D (2021). Effects of cold storage on host *Antheraea pernyi* egg quality for the egg parasitoid *Anastatus fulloi* Sheng and Wang. Insects.

