# Supplementary material for: Cold storage of Gonipterus platensis (Coleoptera: Curculionidae) eggs for Anaphes nitens (Hymenoptera: Mymaridae) rearing
Source: PeerJ. 2026 Mar 13;14:e20903. doi: 10.7717/peerj.20903 (PMC12990896; doi:10.7717/peerj.20903)
Supplement: Supplemental Information 2 [file peerj-14-20903-s002.docx]

Codebook – Categorical Variables

1. Variable: sex

Description: Biological sex of the emerged parasitoid

Codes:

M = Male

F = Female

2. Variable: Repetition

Description: Identifier for experimental replicate.

Codes:

1, 2, 3, ... = Experimental replicates
